# Streamlined, PCR-based testing for *pfhrp2*- and *pfhrp3*-negative *Plasmodium falciparum*

**DOI:** 10.1186/s12936-018-2287-4

**Published:** 2018-04-02

**Authors:** Jonathan B. Parr, Olivia Anderson, Jonathan J. Juliano, Steven R. Meshnick

**Affiliations:** 1 Division of Infectious Diseases, Department of Medicine, University of North Carolina, 130 Mason Farm Rd, Chapel Hill, NC 27599 USA; 2 Department of Epidemiology, Gillings School of Global Public Health, University of North Carolina, 135 Dauer Dr, Chapel Hill, NC 27599 USA; 3 Curriculum in Genetics and Microbiology, University of North Carolina, 321 South Columbia Street, Chapel Hill, NC 27599 USA

**Keywords:** Rapid diagnostic tests, False-negative, Diagnostic resistance, Histidine-rich protein, hrp2, hrp3, RDT, Deletion, Malaria, *Plasmodium falciparum*

## Abstract

**Background:**

Rapid diagnostic tests (RDTs) that detect histidine-rich protein 2 (PfHRP2) are used throughout Africa for the diagnosis of *Plasmodium falciparum* malaria. However, recent reports indicate that parasites lacking the *pfhrp2* and/or histidine-rich protein 3 (*pfhrp3*) genes, which produce antigens detected by these RDTs, are common in select regions of South America, Asia, and Africa. Proving the absence of a gene is challenging, and multiple PCR assays targeting these genes have been described. A detailed characterization and comparison of published assays is needed to facilitate robust and streamlined testing approaches.

**Results:**

Among six *pfhrp2* and *pfhrp3* PCR assays tested, the lower limit of detection ranged from 0.01 pg/µL to 0.1 ng/µL of* P. falciparum* 3D7 strain DNA, or approximately 0.4–4000 parasite genomes/µL. By lowering the elongation temperature to 60 °C, a tenfold improvement in the limit of detection and/or darker bands for all exon 1 targets and for the first-round reaction of a single exon 2 target was achieved. Additionally, assays targeting exon 1 of either gene yielded spurious amplification of the paralogous gene. Using these data, an optimized testing algorithm for the detection of *pfhrp2*- and *pfhrp3*-negative *P. falciparum* is proposed.

**Conclusions:**

Surveillance of *pfhrp2*- and *pfhrp3*-negative *P. falciparum* requires careful laboratory workflows. PCR-based testing methods coupled with microscopy and/or antigen testing serve as useful tools to support policy development. Standardized approaches to the detection of *pfhrp2*- and *pfhrp3*-negative *P. falciparum* should inform efforts to define the impact of these parasites.

**Electronic supplementary material:**

The online version of this article (10.1186/s12936-018-2287-4) contains supplementary material, which is available to authorized users.

## Background

Diagnostic testing is a core component of recent malaria control efforts. In Africa, where the majority of deaths due to malaria occur, rapid diagnostic tests (RDTs) are the most commonly employed malaria diagnostic strategy, accounting for 74% of diagnostic testing among suspected malaria cases [1]. The most commonly used RDTs in Africa rely upon detection of PfHRP2, a *Plasmodium falciparum*-specific antigen expressed by the histidine-rich protein 2 (*pfhrp2*) gene. However, recent reports from select locations in South America, Asia, and Africa of *P. falciparum* parasites lacking *pfhrp2* and/or the histidine-rich protein 3 (*pfhrp3*) gene, which produces an antigen that cross reacts with some PfHRP2-based RDTs, raise concerns about the effectiveness of PfHRP2-based RDTs in affected regions [2–18]. In response, the World Health Organization (WHO) has prioritized efforts to address parasites with deletions of the *pfhrp2* and/or *pfhrp3* (*pfhrp2/3*) genes [19, 20].

The methods required to identify and confirm *pfhrp2/3* gene deletions are challenging, due to the difficulty of proving the absence of a gene. While PCR assays that target *pfhrp2/3* are expected to yield negative results when applied to parasites lacking the gene(s), PCR failure can occur for other reasons. Testing of parasites with intact *pfhrp2/3* genes may yield false-negative results due to DNA concentrations below the assay’s limit of detection, poor quality DNA, variable reagent performance, or other factors.

Cheng et al. published useful guidelines to standardize the reporting of *pfhrp2/3* gene deletions [21]. However, the specific methods employed for the detection and confirmation of deletions continue to vary between laboratories, and recent evidence suggests that atypical elongation temperatures may improve amplification of AT-rich regions of both genes [14, 22]. This manuscript seeks to address these issues by comparing the performance of published PCR assays for *pfhrp2* and *pfhrp3*, exploring the impact of reduced elongation temperatures on assay sensitivity, assessing assay specificity, and describing a streamlined testing algorithm.

## Methods

The performance characteristics of six published PCR assays, including four designed to amplify *pfhrp2* and two designed for *pfhrp3,* were compared [3, 5, 9, 23]. After determining the optimal annealing temperatures for each assay, we assessed their lower limits of detection (LOD) using DNA extracted from cultured *P. falciparum* 3D7 strain parasites. DNA was quantified using the Qubit 2.0 instrument with dsDNA high sensitivity reagents (ThermoFisher Scientific, Waltham, MA) and serially diluted in nuclease-free water to achieve concentrations ranging from 10^−1^ to 10^−7^ ng/µL (seven tenfold dilutions). For each dilution, the assay was performed in triplicate, using different elongation temperatures of 60, 65, and 72 °C. PCR assays were performed on Mastercycler thermocyclers (model AG 22331; Eppendorf, Hamburg, Germany) using 25 µL reaction volumes containing 12.5 µL HotStarTaq Master Mix (Qiagen, Venlo Netherlands), 200–400 nM primers synthesized by Eurofins Genomics (Louisville, KY) with salt-free purification, nuclease-free water, and 3 µL of DNA template (Table 1). For nested reactions, first-round product was diluted 100-fold in nuclease-free water prior to second-round amplification. PCR products were visualized using electrophoresis with 1% agarose gels in TBE buffer (Tris/Borate/EDTA). Finally, LOD testing was repeated using optimized reaction conditions and serial dilutions of 3D7 DNA from a separate stock.

**Table 1:** Published *pfhrp2/3* primer sequences, limits of detection, and optimized conditions

Assay #	Target	Primer sequences (5′→3′)	Reaction conditions	Cycling parameters	LOD,* ng/µL (genomes/µL)	Ref
1	*pfhrp2* (exon 1/2)	Outer: For: GGTTTCCTTCTCAAAAAATAAAG Rev: TCTACATGTGCTTGAGTTTCG Optional—inner: For: GTATTATCCGCTGCCGTTTTTGCC Rev: CTACACAAGTTATTATTAAATGCGGAA	200 nM each primer HotStarTaq MM (Qiagen, Venlo, Netherlands) 3 μL template DNA or 100 × diluted first-round product 25 μL reaction vol	95 °C × 15 min; 40 cycles of 94 °C × 1 min, 50 °C × 1 min for outer primers or 55 °C × 1 min for inner primers, 60 °C × 1 min; 60 °C × 10 min	10^−5^ (~ 0.4)	[3]
2	*pfhrp2* (exons 1/2)	For: TATCCGCTGCCGTTTTTGCC Rev: AGCATGATGGGCATCATCCTA	400 nM each primer HotStarTaq MM 3 μL template DNA 25 μL reaction vol	95 °C × 15 min; 40 cycles of 94 ° × 1 min, 57 °C x 1 min, 60 °C × 1 min; 60 °C × 10 min	10^−1^ (~ 4000)	[9]
3	*pfhrp2* (exon 2)	For: ATTCCGCATTTAATAATAACTTGTGTAGC Rev: ATGGCGTAGGCAATGTGTGG	400 nM each primer HotStarTaq MM 3 μL template DNA 25 μL reaction vol	95 °C × 15 min; 40 cycles of 94 °C × 1 min, 59 °C × 1 min, 72 °C × 1 min; 72 °C × 10 min	10^−4^ (~ 4)	[5]
4	*pfhrp2* (exon 2)	Outer: For: CAAAAGGACTTAATTTAAATAAGAG Rev: AATAAATTTAATGGCGTAGGCA Optional—inner (hemi-nested): For: ATTATTACACGAAACTCAAGCAC Rev: AATAAATTTAATGGCGTAGGCA	400 nM each primer HotStarTaq MM 3 μL template DNA or 100× diluted first-round product 25 μL reaction vol	95 °C × 15 min; 40 cycles of 94 °C × 1 min, 57 °C × 1 min for outer primers or 62 °C for inner primers, 60 °C × 1 min; 60 °C × 10 min	10^−4^ (~ 4)	[23]
5	*pfhrp3* (exons 1/2)	For: TATCCGCTGCCGTTTTTGCTTCC Rev: TGCATGATGGGCATCACCTG	400 nM primers HotStarTaq MM 3 μL template DNA 25 μL reaction vol	95 °C × 15 min; 40 cycles of 94 °C × 1 min, 60 °C × 1 min, 60 °C × 1 min; 60 °C × 10 min	10^−4^ (~ 4)	[9]
6	*pfhrp3* (exon 2)	Outer: For: AATGCAAAAGGACTTAATTC Rev: TGGTGTAAGTGATGCGTAGT Optional—inner (hemi-nested): For: AAATAAGAGATTATTACACGAAAG Rev: TGGTGTAAGTGATGCGTAGT	400 nM primers HotStarTaq MM 3 μL template DNA or 100 × diluted first-round product 25 μL reaction vol	95 °C × 15 min; 40 cycles of 94 °C × 1 min, 55 °C × 1 min, 60 °C × 1 min; 60 °C × 10 min	10^−4^ (~ 4)	[23]
7	*pfldh* (initial qPCR)	For: ACGATTTGGCTGGAGCAGAT Rev: TCTCTATTCCATTCTTTGTCACTCTTTC Probe: FAM-GTAATAGTAACAGCTGGATTTACCAAGGCCCCA-TAMRA	200 nM primers 100 nM probe Probe Master qPCR Mix (Roche Diagnostics, Indianapolis, IN) 2 μL template DNA 12 μL reaction vol	50 °C × 2 min; 95 °C × 10 min; 40 cycles of 95 °C × 15 s, 60 °C × 1 min	10^−4^ (~ 4)	[31]
8	*Pf *-*tubulin* (confirmatory)	For: AATAAATCATAATGATGTGCGCAAGTGATCC Rev: AATAAATCATAATCCTTTGTGGACATTCTTCCTC	300 nM primers FastStart Universal SYBR Green MM (Roche Diagnostics) 3 µL template DNA 25 µL reaction volume	50 °C × 2 min; 95 °C × 10 min; 40 cycles of 95 °C × 15 s, 60 °C × 1 min; Dissociation analysis	10^−3^ (~ 40)	[32, 33]

*LOD* lower limit of detection; *MM* master mix; *Vol* volume; *Bp* base pair

* Typical LOD under the conditions of this laboratory. Assay performance varied between runs, but consistently achieved LODs within one log_10_ of the listed LOD

The specificity of the best performing assays, including those with targets spanning exon 1 and 2 (exon 1/2) and exon 2 alone, was then evaluated. Assays were performed using control DNA from *P. falciparum* Dd2 (MRA-150G) and HB3 (MRA-155G) strain parasites, which lack *pfhrp2* and *pfhrp3,* respectively. Control DNA was obtained from the Malaria Research and Reference Reagent Resource Center ([MR4], BEI Resources, Manassas, Virginia) and diluted to a concentration of 0.1 ng/µL after initial quantification using Qubit as above. For assays that yielded an unexpected result using optimized reaction conditions (i.e. bands from a *pfhrp2* assay performed using *pfhrp2*-deleted Dd2 DNA or bands from a *pfhrp3* assays performed using *pfhrp3*-deleted HB3 DNA), amplicons were sequenced using Sanger sequencing at Eton Bioscience (Research Triangle Park, NC), and assays were repeated at the other elongation temperatures (60, 65, and/or 72 °C). For PCR products with multiple bands appreciated by gel electrophoresis, individual bands were excised, and DNA was extracted using the QIAquick Gel Extraction Kit (Qiagen, Hilden, Germany) before sequencing. Gel extraction was performed according to the manufacturer’s instructions, with the exception of the final DNA elution step, in which we performed two separate elutions using 30 µL aliquots of Buffer EB through the column, followed by a final elution step using the combined 60 µL initial eluate to maximize DNA yield. Raw sequence reads were processed using Sequencher 5.4 (Gene Codes Corporation, Ann Arbor, MI), trimming bidirectional sequences based on confidence values and visual inspection of the chromatograms. We used EMBOSS Water for pairwise nucleotide alignments to the 3D7 (v3.0) *pfhrp2* and *pfhrp3* reference sequences. Sequence identification was based on sequence homology and alignment score, using default settings (DNAfull matrix, gap open penalty 10, gap extend penalty 0.5) [24].

## Results

### Assay performance

Reduced elongation temperatures improved the sensitivity of five of the six assays (Additional file 1: Figures S1, S2). An elongation temperature of 60 °C reduced the LOD by tenfold and/or produced darker bands for all exon 1/2 targets and for the first-round reaction of a single exon 2 target (assay 4). Using optimized elongation temperatures and under our lab conditions, the LOD of published PCR assays for *pfhrp2* and *pfhrp3* varied, with lower limits ranging from approximately 0.01 pg/µL to 0.1 ng/µL of 3D7 DNA, or approximately 0.4–4000 parasite genomes/µL (Table 1). Additionally, while adding a second round of amplification in the best performing nested PCR for *pfhrp2* exon 1/2 (assay 1) resulted in darker bands, the assay’s LOD was unchanged.

### Amplification of paralogous genes by exon 1/2 assays

**Fig. 1:**
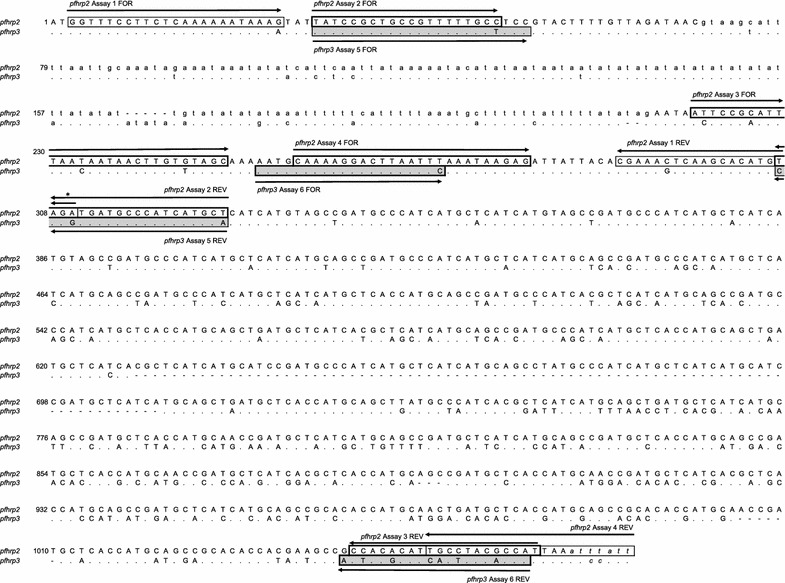
*Pfhrp2* and *pfhrp3* gene sequence homology. Alignment of reference sequences from the consensus 3D7 (v3.0) genome, 5′→3′, with expected binding sites for *pfhrp2* assays (white boxes) and *pfhrp3* assays (gray boxes). The reverse primer sequence for Assay 2 includes an a single-base insertion (cytosine) at the location indicated by an asterisk (*). Identical bases are indicated by a period (.), missing bases by a dash (-), substitutions by the discordant base, and non-coding regions by lower case font

Unexpectedly, paralogous amplification by assays targeting exon 1/2 but not exon 2 of both genes was observed. Bands were visualized from assays targeting exon 1/2 of *pfhrp2* (assay 1) using Dd2 (*pfhrp2*-deleted) control DNA (Additional file 1: Figure S3) and those targeting exon 1/2 of *pfhrp3* (assay 5) using HB3 (*pfhrp3*-deleted) control DNA (Additional file 1: Figure S4), respectively. Assay 1 produced unexpected bands at all three extension temperatures tested, while assay 5 produced bands when tested using 60 and 65 °C extension temperatures but not at 72 °C. All tested exon 2 assays for both genes (assay 3, 4, and 6) produced negative results using Dd2 or HB3 DNA, as expected. *Pfhrp2* exon 1/2 assay 2 was not tested due to its poor performance during initial LOD testing, presumably a result of a single base insertion near the 3′ end of the reverse primer compared to the 3D7 reference sequence. The sequence homology of *pfhrp2* and *pfhrp3* exon 1/2 primer binding sites (Fig. 1) suggested that amplification of paralogous genes had occurred—i.e., the amplicons generated by the *pfhrp2* exon 1/2 assay using Dd2 strain control DNA represented *pfhrp3* amplification and that those generated by the *pfhrp3* exon 1/2 assay using HB3 strain control DNA represented *pfhrp2* amplification.

Sequencing results confirmed amplification of *pfhrp3* by the *pfhrp2* exon 1/2 assay, and vice versa. With Dd2 strain (*pfhrp2*-deleted) template, the *pfhrp2* exon 1/2 assay (assay 1) unexpectedly produced a single band with a fragment length of approximately 300 bp. The amplicon’s sequence aligned to the *pfhrp3* gene with 92% sequence homology (Additional file 1: Figure S5). With HB3 strain (*pfhrp3*-deleted) template, the *pfhrp3* exon 1/2 assay (assay 5) unexpectedly produced two clear bands with fragment lengths of approximately 300 and 800 bp and a faint band at approximately 400 bp. Sequences generated using DNA extracted from each band aligned to the *pfhrp2* gene, with 98, 99, and 97% sequence homology for the 300, 400, and 800 bp fragments, respectively (Additional file 1: Figure S6). However, when applied to 3D7 strain (*pfhrp2/3*-positive) control template, both exon 1/2 assays produced the expected result: a single band with a sequence that aligned to *pfhrp2* with 96% homology or *pfhrp3* with 99% sequence homology (for assays 1 and 5, respectively).

### Streamlined testing algorithm

**Fig. 2:**
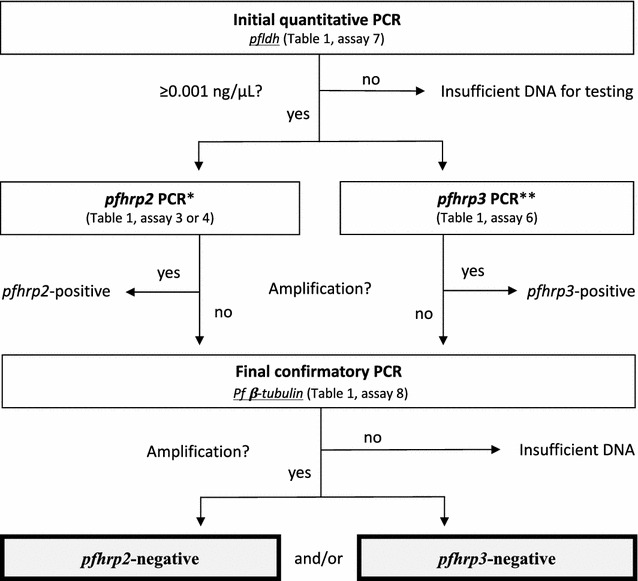
Proposed testing pipeline. Single-step assays are favoured to reduce the risk of contamination, and assays should be performed in duplicate. In addition to positive *P. falciparum* (e.g. 3D7 strain) DNA and no template controls, either *pfhrp2-negative (e.g. Dd2 strain) or **pfhrp3-negative (e.g. HB3 strain) *P. falciparum* DNA controls should be used

These findings were used to develop a streamlined, PCR-based testing pipeline for *pfhrp2/3*-negative *P. falciparum* (Fig. 2) that incorporates optimized elongation temperatures, LOD testing results, and assay specificity.

## Discussion

By lowering the LOD and employing assays that distinguish *pfhrp2* from *pfhrp3*, this testing algorithm provides an improved approach to PCR-based detection of *pfhrp2/3*-negative *P. falciparum*. Importantly, PCR-based approaches for identification of *pfhrp2*- and *pfhrp3*-negative parasites must be coupled with verification of *P. falciparum* parasitaemia and confirmation that parasite DNA is present at concentrations above the LODs of the *pfhrp2* and *pfhrp3* assays. These goals were achieved by employing assays targeting two *P. falciparum*-specific, single-copy genes, lactate dehydrogenase (*pfldh*) and *P. falciparum beta tubulin (PfBtubulin)*, as the initial and final steps of the testing pipeline.

Lowering the elongation temperature improved the LOD of all published assays with exon 1/2 targets on either gene. This finding likely represents improved amplicon extension across the AT-rich intron between the exons as suggested by previous reports [22]. Unexpected amplification of paralogous gene targets by the *pfhrp2* and *pfhrp3* exon 1/2 assays was observed, presumably due to sequence homology at the primer binding sites. In regions where co-existing *pfhrp2* and *pfhrp3* deletions are common, the impact of non-specific amplification is expected to be reduced [25–27]. Additionally, the absence of paralogous amplification of 3D7 control DNA suggests that the availability of abundant, completely homologous primer binding sites early in PCR cycling reduces the likelihood of exponential amplification after mispriming. To reduce the risk of unintentional amplification of paralogous genes, this testing algorithm uses assays targeting exon 2 of both genes. This approach also permits analysis of the repetitive sequences that encode epitopes recognized by anti-PfHRP2 antibodies [28].

A broad range of LOD results was observed for published *pfhrp2* and *pfhrp3* assays, spanning over 4 orders of magnitude under the laboratory conditions employed during this study. These differences were addressed in the resulting testing pipeline (Fig. 2) by defining an initial threshold DNA concentration tenfold higher than the LOD of the downstream *pfhrp2* and *pfhrp3* assays. In addition, a stringent, final single-copy-gene PCR that meets the same LOD requirement was included, providing confirmation that sample degradation has not occurred during the testing process. The typical workflow employed in this laboratory includes assays 3 and 6 for *pfhrp2* and *pfhrp3* testing, respectively, performed in duplicate. For discordant results (i.e. one of two replicates positive), samples are called positive if there is a clear band of appropriate fragment length. If not, the assay is repeated, and the final call is based on the third result. Because the first-round of the nested assays achieved LODs below the initial and final confirmatory, falciparum-specific assays, their use as single-step assays is favoured in this laboratory to reduce the risk of contamination and improve work flow.

In settings where real-time PCR is not feasible, the proposed initial lactate dehydrogenase (*pfldh*) quantitative PCR assay and the final confirmatory *P. falciparum beta tubulin (PfBtubulin)* assays could be replaced with traditional PCR assays with LODs above the LOD of the *pfhrp2* and *pfhrp3* assays. Because assay performance can vary from laboratory to laboratory and with different reagents or equipment, it is essential to confirm the LOD of each assay using the reagents and laboratory infrastructure at hand.

In addition to PCR-based testing, current guidelines recommend independent confirmation of *P. falciparum* parasitaemia using microscopy or a non-PfHRP2-based RDT, such as an RDT that detects *P. falciparum* lactate dehydrogenase (pf-pLDH), before making deletion calls [19, 21]. Quantification of circulating PfHRP2 antigen is also a valuable tool that can be particularly useful for assessing PfHRP2-RDT-negative but *pfhrp2/3*-PCR-positive isolates with impaired protein expression [29]. Additionally, novel assays under development such as those targeting regionally specific deletion breakpoints or employing droplet digital PCR, have potential to improve throughput [30].

## Conclusions

Surveillance of *pfhrp2*- and *pfhrp3*-negative *P. falciparum* requires careful laboratory workflows. PCR-based testing methods, coupled with microscopy and/or antigen testing, serve as useful tools to support policy development. Standardized approaches to the detection of *pfhrp2*- and *pfhrp3*-negative *P. falciparum* should inform efforts to define the impact of these parasites [20, 21].

## Additional files

**Additional file 1: Figure S1.** *Pfhrp2* assay performance using serially diluted *P. falciparum* 3D7 strain DNA. Elongation temperatures were varied as listed below. All other reaction conditions are specified in Table 1. **Figure S2.** *Pfhrp3* assay performance using serially diluted *P. falciparum* 3D7 strain DNA. Elongation temperatures were varied as listed below. All other reaction conditions are specified in Table 1. **Figure S3.** Representative agarose gel electrophoresis depicting unexpected spurious bands from Dd2 strain (*pfhrp2*-deleted) control DNA. PCR targeting *pfhrp2* exon 1/2 (assay 1 outer) yielded a spurious ~ 300 bp band from serial dilutions of *pfhrp2*-deleted Dd2 strain control DNA at all three elongation temperatures. **Figure S4.** Representative agarose gel electrophoresis depicting unexpected spurious bands from HB3 strain (*pfhrp3*-deleted) control DNA. PCR targeting *pfhrp3* exon 1/2 (assay 5) yielded spurious bands at ~ 300, 400, and 800 bp from serial dilutions of Dd2 strain control DNA using optimized elongation temperatures (Table 1). **Figure S5.** *Pfhrp3* assay performance using serially diluted *P. falciparum* 3D7 strain DNA. Elongation temperatures were varied as listed below. All other reaction conditions are specified in Table 1. The sequence of *pfhrp2* exon 1/2 (assay 1) PCR product aligns to *pfhrp3,* due to spurious PCR amplification of the Dd2 *pfhrp3* gene. PCR was performed using 0.01 ng/μL of 3D7 (*pfhrp2*-positive) and Dd2 (*pfhrp2*-negative) control DNA, respectively (see Additional file 1: Figure S3), followed by Sanger sequencing of amplicons. Reference sequences from the consensus 3D7 (v3.0) genome for *pfhrp2* and *pfhrp3* are displayed on the top two rows (REF), from 5′→ 3′, with capital letters for coding regions and genetic coordinates in reference to the *pfhrp2* gene. Identical bases are indicated by a period (.), missing bases by a dash (-), substitutions by the discordant base. PCR product sequence contigs are highlighted as follows: 3D7 control DNA (light gray); Dd2 control DNA (dark gray). **Figure S6.** The sequences of *pfhrp3* exon 1/2 (assay 5) PCR product align to *pfhrp2*, due to spurious PCR amplification of the HB3 *pfhrp2* gene. PCR was performed using 0.01 ng/μL of 3D7 (*pfhrp3*-positive) and HB3 (*pfhrp3*-negative) control DNA, respectively (see Additional file 1: Figure S4), followed by Sanger sequencing of amplicons. Reference sequences from the consensus 3D7 (v3.0) genome for *pfhrp2* and *pfhrp3* are displayed on the top two rows (REF), from 5′→ 3′, with capital letters for coding regions and genetic coordinates in reference to the *pfhrp3* gene. Identical bases are indicated by a period (.), missing bases by a dash (-), substitutions by the discordant base. PCR product sequence contigs are highlighted as follows: 3D7 control DNA (medium gray) and HB3 control DNA 300 bp fragment (light gray), 400 bp fragment (medium gray), and 800 bp fragment (dark gray)

**Additional file 2.** FASTA file containing Sanger sequences of amplicons produced by* pfhrp2/3* exon 1/2 assays when applied to Dd2 (*pfhrp2*-negative), HB3 (*pfhrp3*-negative), and 3D7 (*pfhrp2/3*-positive) DNA
