# Supplementary material for: Streamlined, PCR-based testing for pfhrp2- and pfhrp3-negative Plasmodium falciparum
Source: Malar J. 2018 Apr 2;17:137. doi: 10.1186/s12936-018-2287-4 (PMC5879555; doi:10.1186/s12936-018-2287-4)

SUPPLEMENTARY MATERIAL

Figure S1 – *Pfhrp2* assay performance using serially diluted *P. falciparum* 3D7 strain DNA. Elongation temperatures were varied as listed below. All other reaction conditions are specified in Table 1.

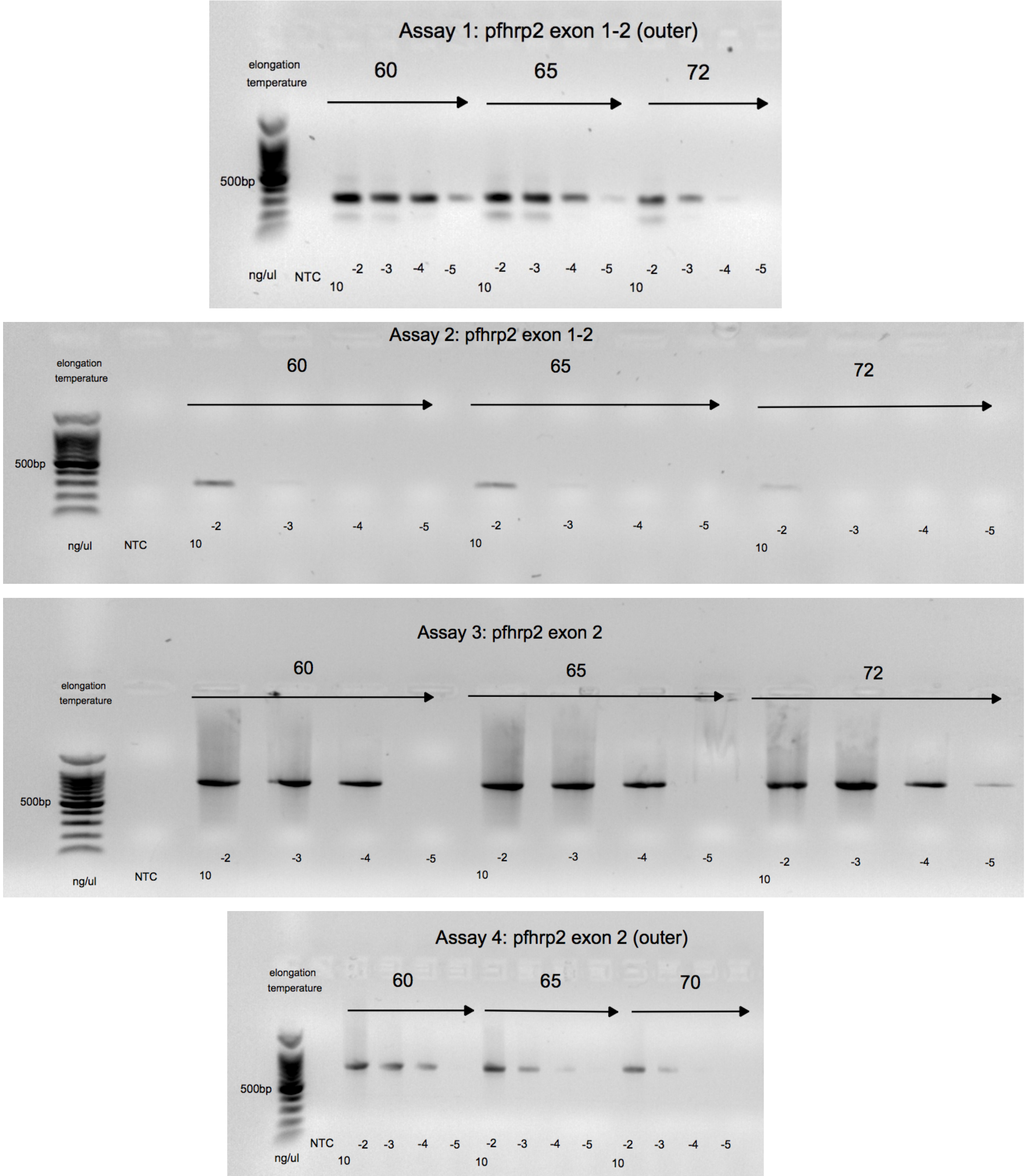

https://doi.org/10.1186/s12936-018-2287-4

Figure S2 – *Pfhrp3* assay performance using serially diluted *P. falciparum* 3D7 strain DNA. Elongation temperatures were varied as listed below. All other reaction conditions are specified in Table 1.

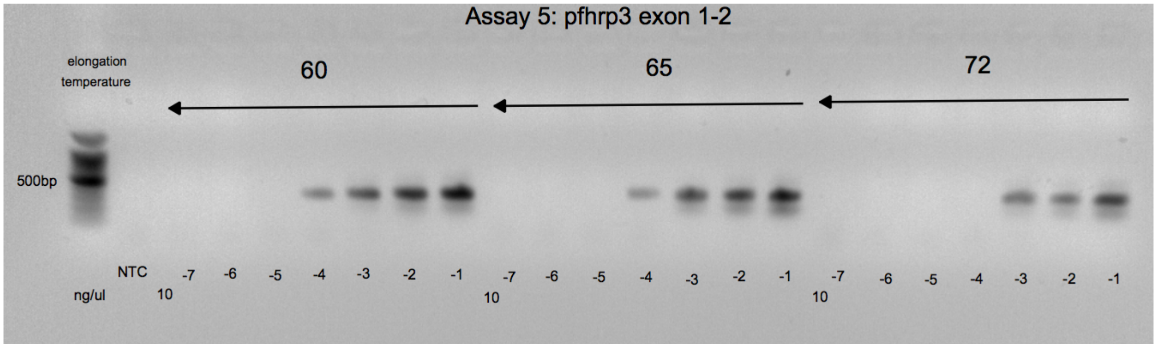

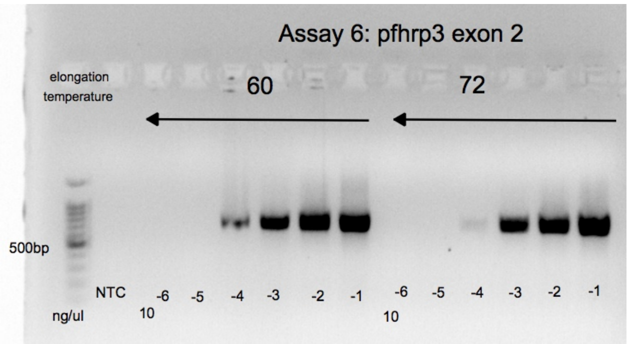

https://doi.org/10.1186/s12936-018-2287-4

Figure S3 – Representative agarose gel electrophoresis depicting unexpected spurious bands from Dd2 strain (*pfhrp2*-deleted) control DNA. PCR targeting *pfhrp2* exon 1/2 (assay 1 outer) yielded a spurious ~300bp band from serial dilutions of *pfhrp2*-deleted Dd2 strain control DNA at all three elongation temperatures.

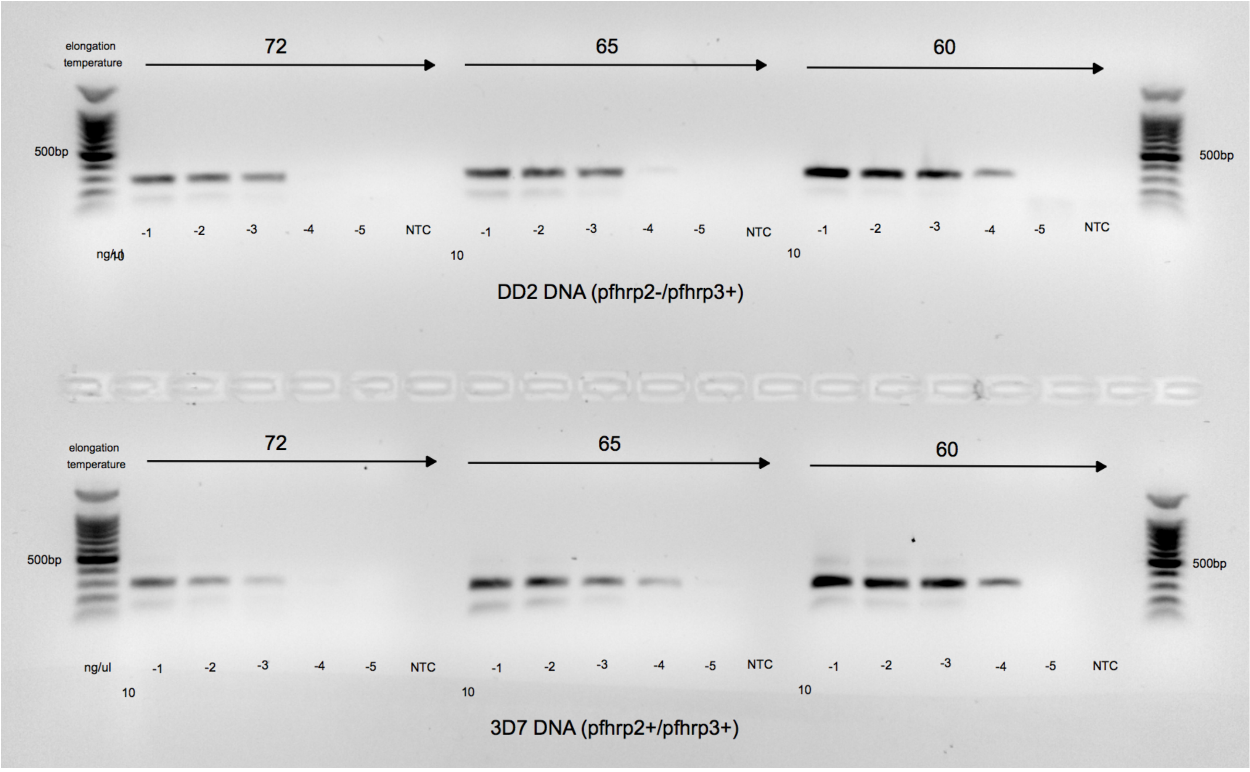

Figure S4 – Representative agarose gel electrophoresis depicting unexpected spurious bands from HB3 strain (*pfhrp3*-deleted) control DNA. PCR targeting *pfhrp3* exon 1/2 (assay 5) yielded spurious bands at ~300, 400, and 800bp from serial dilutions of Dd2 strain control DNA using optimized elongation temperatures (Table 1).

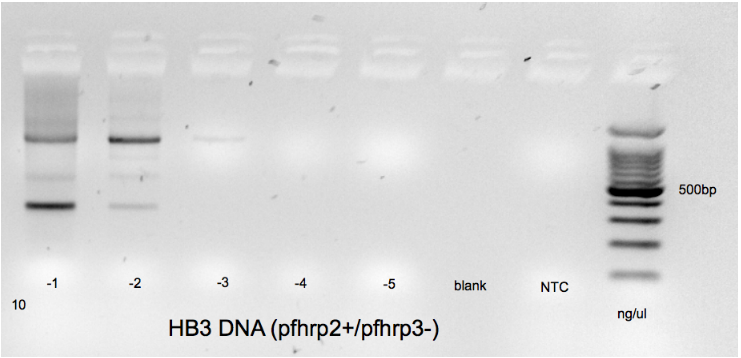

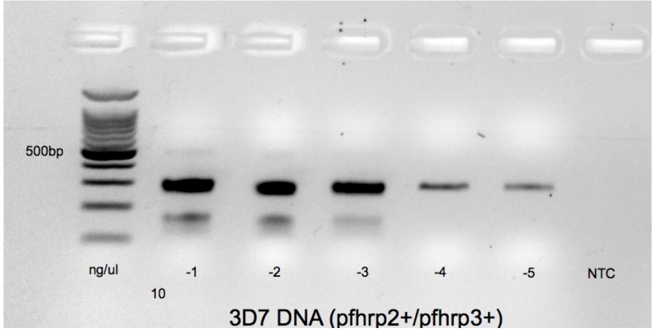

https://doi.org/10.1186/s12936-018-2287-4

Figure S5 – The sequence of *pfhrp2* exon 1/2 (assay 1) PCR product aligns to *pfhrp3,* due to spurious PCR amplification of the Dd2 *pfhrp3* gene. PCR was performed using 0.01ng/μL of 3D7 (*pfhrp2*-positive) and Dd2 (*pfhrp2*-negative) control DNA, respectively (see Figure S3), followed by Sanger sequencing of amplicons. Reference sequences from the consensus 3D7 (v3.0) genome for *pfhrp2* and *pfhrp3* are displayed on the top two rows (REF), from 5'->3', with capital letters for coding regions and genetic coordinates in reference to the *pfhrp2* gene. Identical bases are indicated by a period (.), missing bases by a dash (-), substitutions by the discordant base. PCR product sequence contigs are highlighted as follows: 3D7 control DNA (light gray); Dd2 control DNA (dark gray).

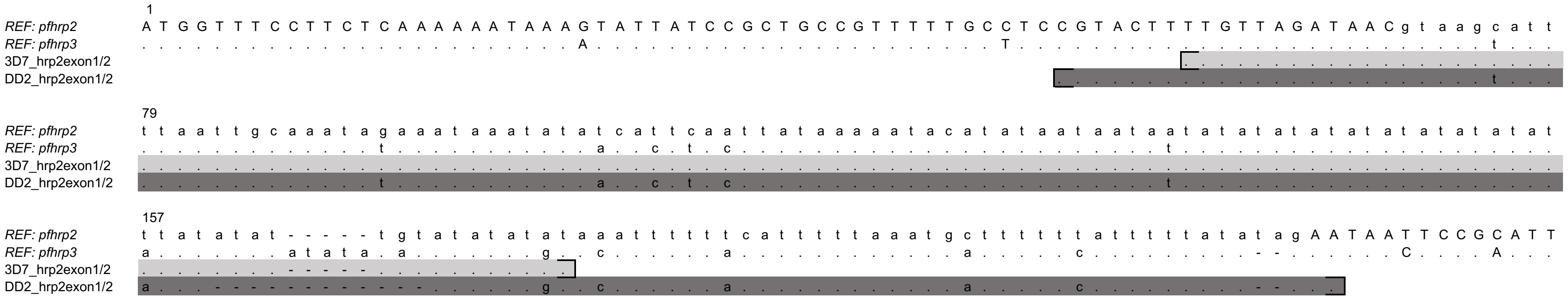

Figure S6 – The sequences of *pfhrp3* exon 1/2 (assay 5) PCR product align to *pfhrp2*, due to spurious PCR amplification of the HB3 *pfhrp2* gene. PCR was performed using 0.01ng/µL of 3D7 (*pfhrp3*-positive) and HB3 (*pfhrp3*-negative) control DNA, respectively (see Figure S4), followed by Sanger sequencing of amplicons. Reference sequences from the consensus 3D7 (v3.0) genome for *pfhrp2* and *pfhrp3* are displayed on the top two rows (REF), from 5'->3', with capital letters for coding regions and genetic coordinates in reference to the *pfhrp3* gene. Identical bases are indicated by a period (.), missing bases by a dash (-), substitutions by the discordant base. PCR product sequence contigs are highlighted as follows: 3D7 control DNA (medium gray) and HB3 control DNA 300bp fragment (light gray), 400bp fragment (medium gray), and 800bp fragment (dark gray).

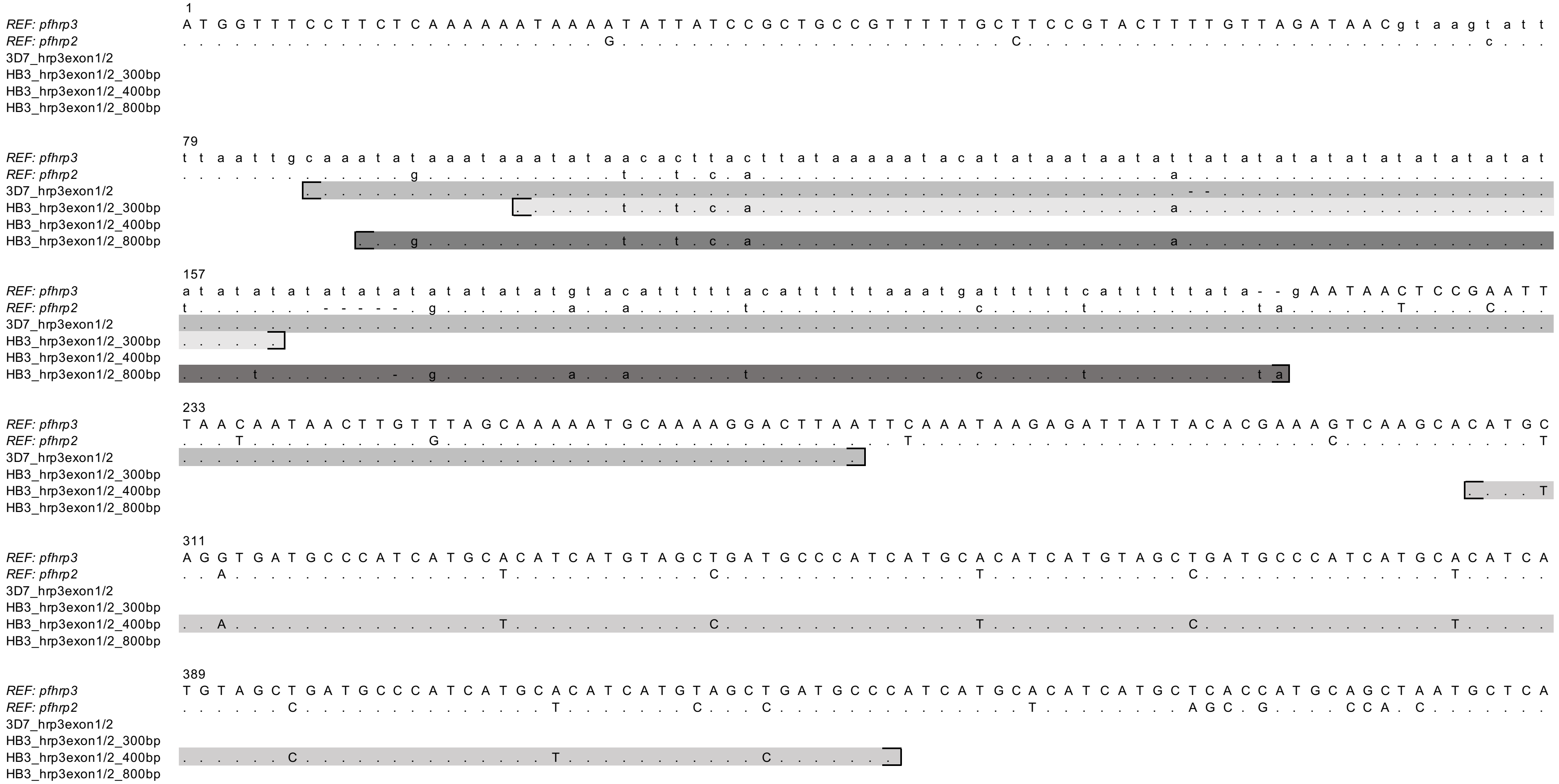

Supplement: Supplementary file 1 — Additional file 1: Figure S1. Pfhrp2 assay performance using serially diluted P. falciparum 3D7 strain DNA. Elongation temperatures were varied as listed below. All other reaction conditions are specified in Table 1. Figure S2. Pfhrp3 assay performance using serially diluted P. falciparum 3D7 strain DNA. Elongation temperatures were varied as listed below. All other reaction conditions are specified in Table 1. Figure S3. Representative agarose gel electrophoresis depicting unexpected spurious bands from Dd2 strain (pfhrp2-deleted) control DNA. PCR targeting pfhrp2 exon 1/2 (assay 1 outer) yielded a spurious ~ 300 bp band from serial dilutions of pfhrp2-deleted Dd2 strain control DNA at all three elongation temperatures. Figure S4. Representative agarose gel electrophoresis depicting unexpected spurious bands from HB3 strain (pfhrp3-deleted) control DNA. PCR targeting pfhrp3 exon 1/2 (assay 5) yielded spurious bands at ~ 300, 400, and 800 bp from serial dilutions of Dd2 strain control DNA using optimized elongation temperatures (Table 1). Figure S5. Pfhrp3 assay performance using serially diluted P. falciparum 3D7 strain DNA. Elongation temperatures were varied as listed below. All other reaction conditions are specified in Table 1. The sequence of pfhrp2 exon 1/2 (assay 1) PCR product aligns to pfhrp3, due to spurious PCR amplification of the Dd2 pfhrp3 gene. PCR was performed using 0.01 ng/μL of 3D7 (pfhrp2-positive) and Dd2 (pfhrp2-negative) control DNA, respectively (see Additional file 1: Figure S3), followed by Sanger sequencing of amplicons. Reference sequences from the consensus 3D7 (v3.0) genome for pfhrp2 and pfhrp3 are displayed on the top two rows (REF), from 5′→ 3′, with capital letters for coding regions and genetic coordinates in reference to the pfhrp2 gene. Identical bases are indicated by a period (.), missing bases by a dash (-), substitutions by the discordant base. PCR product sequence contigs are highlighted as follows: 3D7 control DNA (light g [file 12936_2018_2287_MOESM1_ESM.pdf]
